# NHE8 Is Essential for RPE Cell Polarity and Photoreceptor Survival

**DOI:** 10.1038/srep09358

**Published:** 2015-03-20

**Authors:** Chun-hong Xia, Haiquan Liu, Debra Cheung, Felicia Tang, Bo Chang, Mei Li, Xiaohua Gong

**Affiliations:** 1 School of Optometry and Vision Science Program, University of California, Berkeley, Berkeley, CA 94720, USA; 2 The Jackson Laboratory, Bar Harbor, ME 04609, USA

## Abstract

A new N-ethyl-N-nitrosourea (ENU)-induced mouse recessive mutation, identified by fundus examination of the eye, develops depigmented patches, indicating retinal disorder. Histology data show aberrant retinal pigment epithelium (RPE) and late-onset photoreceptor cell loss in the mutant retina. Chromosomal mapping and DNA sequencing reveal a point mutation (T to A) of the *Slc9a8* gene, resulting in mutant sodium/proton exchanger 8 (NHE8)-M120K protein. The lysine substitution decreases the probability of forming the 3^rd^ transmembrane helix, which impairs the pore structure of the Na^+^/H^+^ exchanger. Various RPE defects, including mislocalization of the apical marker ezrin, and disrupted apical microvilli and basal infoldings are observed in mutant mice. We have further generated NHE8 knockout mice and confirmed similar phenotypes, including abnormal RPE cells and late-onset photoreceptor cell loss. Both *in vivo* and *in vitro* data indicate that NHE8 co-localizes with ER, Golgi and intracellular vesicles in RPE cells. Thus, NHE8 function is necessary for the survival of photoreceptor cells and NHE8 is important for RPE cell polarity and function. Dysfunctional RPE may ultimately lead to photoreceptor cell death in the NHE8 mutants. Further studies will be needed to elucidate whether or not NHE8 regulates pH homeostasis in the protein secretory pathways of RPE.

Vision depends on the photoreceptor cells of the retina to catch photons to start the visual process^1^. The retinal pigment epithelium (RPE), adjoining the outer segments (OS) of photoreceptor cells in the back of the retina, is essential for maintaining the homeostasis and survival of photoreceptor cells^2^,^3^. Photoreceptor cell death in age-related macular degeneration (AMD) is the leading cause of untreatable blindness in industrialized countries^4^,^5^. The fundamental molecular and cellular mechanisms that regulate and maintain life-long function of the RPE and photoreceptor cells are still not well understood^6^. Understanding these mechanisms is critical for elucidating the events that trigger pathological cascades towards photoreceptor cell death, which occurs in human eye diseases such as retinitis pigmentosa (RP) and AMD^5^,^7^,^8^.

RPE is a monolayer of polarized cells that plays many essential roles in the maintenance and homeostasis of photoreceptor cells^9^. The apical ends of RPE abut and engulf photoreceptor OS through phagocytosis whereas the basolateral sides lie on Bruch's membrane and transport nutrients, digested metabolic wastes, ions and water between the retina and the choroidal vasculature in the back of the eye. RPE provides the recycle of retinoids for the phototransduction pathway as well as the blood-retinal barriers. Due to phototoxicity, the daily renewal of photoreceptor OS is profoundly important to the survival of photoreceptors and maintenance of retinal health^10^. In the last decade, significant progress has been made in understanding some of the mechanisms that control RPE phagocytosis, especially OS recognition and engulfment signaling molecules including the Gas6/MerTK pathway^3^,^11^. Late stage of phagocytosis is the recycling and degradation mediated by the endosome/lysosome pathway^12^,^13^; the underlying mechanisms that determine the recycling and degradation of proteins in RPE are still not well understood^12^,^14^. Specific markers for polarized RPE cells have been identified for their unique functions in the RPE^15^. However, mechanisms that control and maintain RPE polarity and function also require further investigation^6^,^15^. In mice, photoreceptor cells start to form OS at postnatal day 10^16^; RPE establishes cell polarity after birth and phagocytosis occurs at around the age of two weeks when mice open eyes^17^,^18^.

From a forward genetic study of new retinal degeneration mouse mutations, we have found that mutations of the SLC9A8 gene, which encodes the sodium/proton exchanger 8 (NHE8), lead to slow photoreceptor cell death. NHE8 is a member of the solute carrier family 9 (SLC9). Sodium/proton exchangers (NHEs) are a large group of monovalent cation/proton antiporters that predominately move Na^+^ in exchange for H^+^, and are involved in diverse physiological processes including the regulation of intracellular pH, absorption of sodium into epithelia, salt tolerance, cell volume, cell adhesion, cell proliferation, organelle biogenesis, and protein trafficking^19^. NHEs can be divided into two subfamilies based on protein cellular localization and sequence alignments; one is a cell surface subfamily, including mammalian NHEs 1–5, and the other is an intracellular subfamily localized to organelle membranes, including mammalian NHEs 6–9^20^,^21^. NHE8 belongs to the intracellular subfamily, and previous studies have reported that NHE8 is expressed on the apical membrane of intestinal epithelial cells to prevent infectious bacterial adherence^22^. Our current work suggests that NHE8 is essential for the survival of photoreceptor cells and plays an important role in the retina by regulating RPE cell polarity and function.

## Results

**Figure 1:**
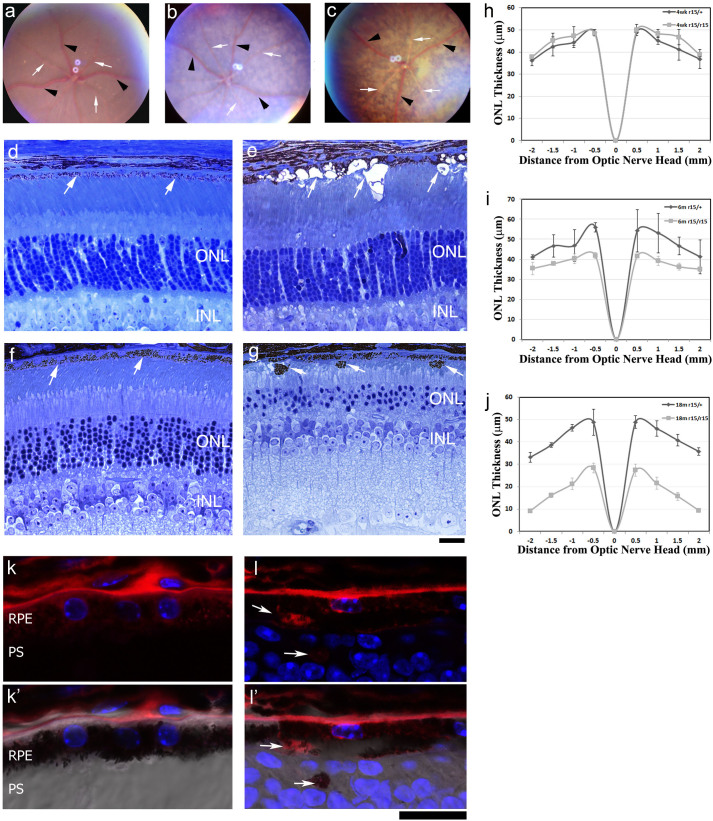
The *r15* mutant mice display late-onset recessive retinal degeneration with aberrant RPE cells. Fundus photos from a 4-week old *r15* heterozygous mouse (a), a 4-week old *r15* homozygous mutant mouse (b) and a 6-month old homozygous mutant mouse (c); retinal veins are indicated by black arrowheads and retinal arteries indicated by white arrows. Toluidine blue stained retinal sections from 4-week old heterozygous (d) and homozygous (e) mutant mice, and 18-month old heterozygous (f) and homozygous (g) mutant mice; white arrows indicate RPE; ONL, outer nuclear layer; INL, inner nuclear layer. Spider graphs show ONL thickness in *r15* heterozygous mice (black lines, n = 3) and *r15* homozygous mice (grey lines, n = 3) at the ages of 4 weeks (h), 6 months (i), and 18 months (j). Immunostaining of 20-month old *r15* heterozygous (k) and homozygous (l) retinal frozen sections indicate that mislocalized pigmented structures are labeled by an anti-RPE65 antibody (red, white arrows); nuclei are labeled with Dapi (blue); differential interference contrast (DIC) merged images (k′ and l′) are underneath the corresponding images; RPE, retinal pigment epithelium; PS, photoreceptor segments; Scale bars for panels d-g and k-l′, 20 μm.

The *r15* mutation, identified from a fundus screen of ENU-induced mutant mice, displayed a recessive retinal degeneration^23^. At the age of 4 weeks, compared to the normal fundus image of heterozygous mice (r15/+) (Fig. 1a), homozygous mutant mice (r15/r15) showed depigmented patches (Fig. 1b). More severe depigmentation was observed in aged mutant mice (Fig. 1c). Histological sections revealed that the number of outer nuclear layers (ONL) in heterozygous mice was around 10–12 without noticeable changes from the ages of 4 weeks to 18 months (Fig. 1d,f). In contrast, 4-week old homozygous mutant mice displayed aberrant RPE cells with vacuole-like structures while the number of ONL remained comparable to the control at about 10–12 layers (Fig. 1e). The loss of photoreceptor cells was observed in homozygous mutant mice only after the age of 6 weeks. The ONL decreased to 6–8 layers in 6-month old homozygous mutant mice (data not shown) and about 3–4 layers in 18-month old homozygous mutant mice, which also had pigmented aberrant cells submerged in the subretinal space (Fig. 1g). We measured the ONL thickness of the heterozygous and homozygous mutant retinas from mice at the ages of 4 weeks, 6 months and 18 months (Fig. 1h,i,j). At 4 weeks of age, there is no significant difference in ONL thickness between the *r15* heterozygous and homozygous mice (p > 0.5); however, significantly reduced ONL thickness was observed in 6-month old (p < 0.05, except the −2 mm data point) and 18-month old homozygous (p < 0.001) mutant mice compared to the heterozygous controls. To confirm the pigmented aberrant structures are aberrant RPE cells, immunostaining was performed in 20-month old retinal sections. Compared to normal RPE65 positive RPE cells in the control heterozygous retina (Fig. 1k,k′), RPE65 positive aberrant pigmented structures were observed in the photoreceptor cell layer in the homozygous mutant (Fig. 1l,l′). Thus, depigmentation in fundus images likely results from pathological changes of RPE in *r15* homozygous mutant retinas; dysfunctional RPE might ultimately contribute to the late-onset photoreceptor loss.

**Figure 2:**
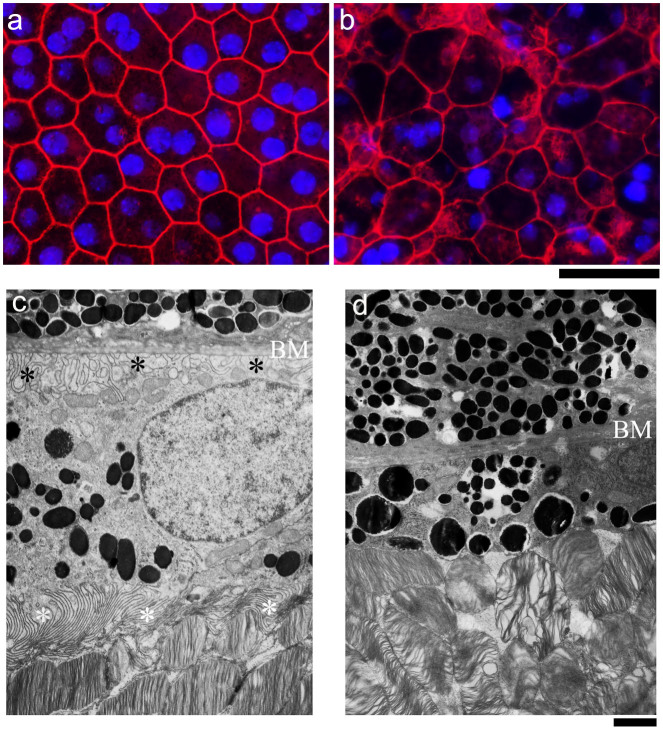
Morphologic changes of RPE caused by the *r15* mutation. Rhodamine-phalloidin (for F-actin, red) and Dapi (for nuclei, blue) double labeling of whole mount RPEs from 4-week old wild-type (a) and *r15* homozygous mutant (b) mice. Electron micrographs of RPEs from 5-month old wild-type (c) and *r15* homozygous mutant retinas (d); white asterisks indicate apical microvilli and black asterisks indicate basal infoldings near Bruch's membrane (BM); note the absence of distinct basal infoldings and apical microvilli in the mutant retina (d). Scale bars for panels a and b, 20 μm; scale bars for panels c and d, 1 μm.

We further investigated the cellular defects of RPE in the *r15* mutant mice. Double labeled images of rhodamine-phalloidin and DAPI revealed a typical hexagonal array of RPE cells containing single or double nuclei, with a sharp F-actin network at cell boundaries in the whole mount RPE of a 4-week old wild-type sample (Fig. 2a). In contrast, an age-matched *r15* homozygous mutant displayed disorganized RPE cells with irregular shape and size and disrupted F-actin network at some cell boundaries (Fig. 2b); moreover, the *r15* mutant RPE flat-mount image was fuzzy, reflecting substantial defects and changes of RPE cells observed by histology analysis (Fig. 1e). Electron microscope images showed abundant microvilli at the apical surface next to organized outer segments of photoreceptors as well as typical basal infoldings next to the Bruch's membrane in 5-month old wild-type RPE cells (Fig. 2c); however, RPE from 5-month old homozygous *r15* mice had neither apical microvilli nor distinct basal infoldings (Fig. 2d). These morphological data indicate that *r15* mutant RPE cells lose polarity and lack the distinct structures and organization that are needed for performing functions such as phagocytosis. Therefore, dysfunctional RPE likely causes the late-onset death of photoreceptor cells in *r15* mutant mice.

**Figure 3:**
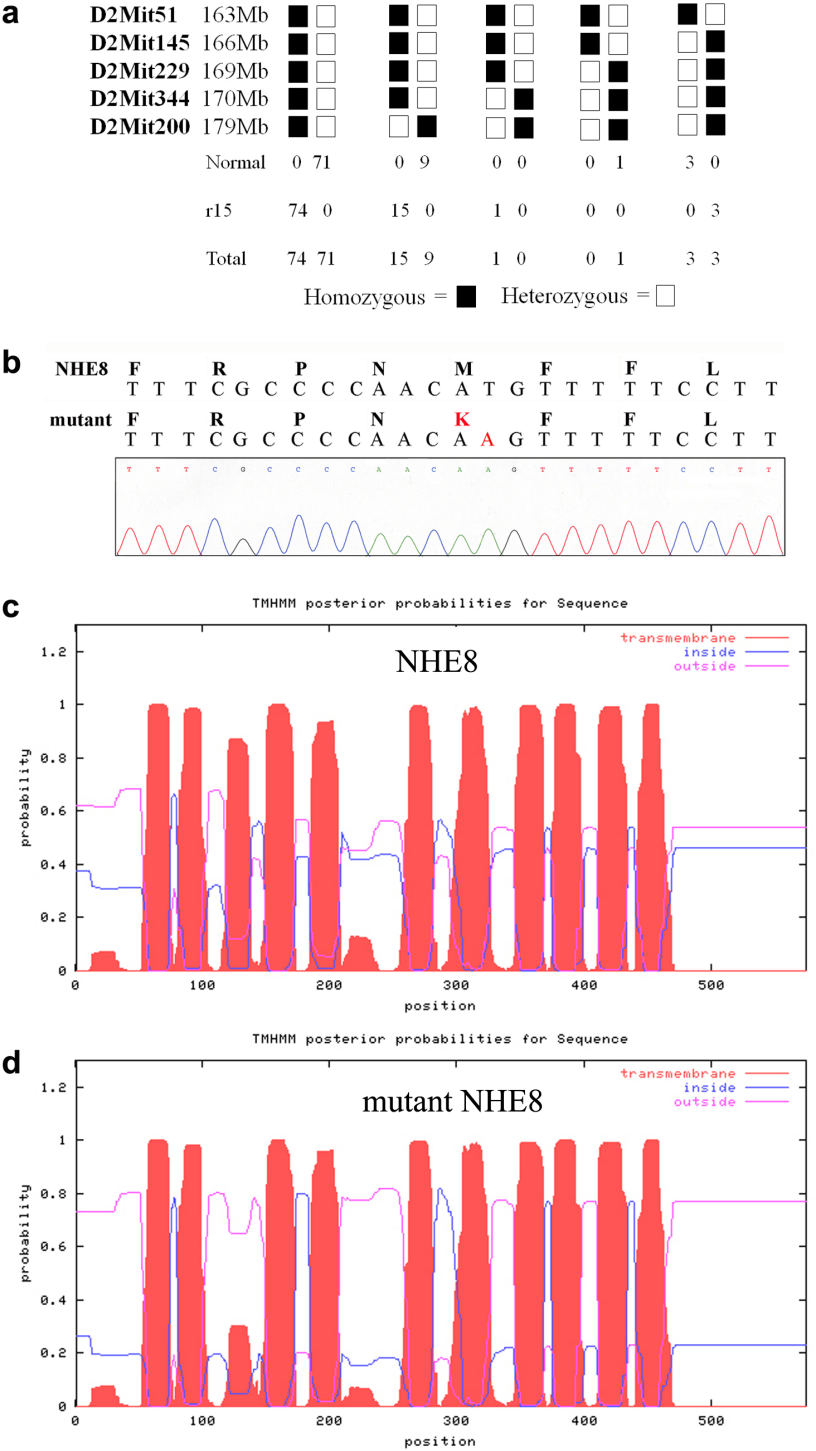
Identification of the causative gene, *Slc9a8,* for the r15 mutation. (a) Linkage analysis of the *r15* mutation was based on the phenotypes and genotypes of 177 meioses. The mutation was mapped to a 6 Mb region between markers D2Mit51 and D2Mit229 on mouse chromosome 2. Positions of the markers are based on the Ensembl Mouse Genome Server. (b) DNA sequencing data revealed a missense (T to A) mutation of the *Slc9a8* gene, resulting in a substitution of methionine (M) by lysine (K) at the 120^th^ amino acid of the NHE8 protein in the *r15* mutation. (c) Wild-type NHE8 protein was predicted to form 11 transmembrane helices by the program TMHMM (v. 1.0) (http://www.cbs.dtu.dk/). (d) NHE8-M120K mutant protein decreased the probability of forming the 3^rd^ transmembrane helix containing residue 120.

To identify the causative gene, we performed a genome-wide linkage analysis. The *r15* mutation was mapped to mouse chromosome 2. Based on linkage data from genomic DNA samples of 177 meioses, we further mapped this mutation into a 6 Mb interval region between the markers D2Mit51 and D2Mit229 on the Ensembl Mouse Genome Server (Fig. 3a). Sequencing data revealed that the *r15* retinal phenotype was correlated with a missense mutation (T to A) of the *Slc9a8* gene, which results in substitution of methionine (M) by lysine (K) at residue 120 of the NHE8 protein (Fig. 3b), mutant NHE8-M120K. The probability of forming transmembrane helices predicted by the program TMHMM (v. 1.0) (http://www.cbs.dtu.dk/) suggested that wild-type NHE8 contained 11 transmembrane helices (Fig. 3c) but mutant NHE8-M120K abolished the 3^rd^ transmembrane helix (Fig. 3d). Thus, mutant NHE8-M120K with a disrupted 3^rd^ transmembrane helix likely impairs its function as a sodium-proton exchanger in the RPE. According to standard genetic nomenclature guidelines, the *r15* mouse mutant line should be named *Slc9a8^r15(M120K/M120K)^*. To be consistent with previous studies of sodium-hydrogen exchangers in the literature, we use NHE8-M120K in the paper to describe this mutant mouse line.

To confirm that a dysfunctional NHE8-M120K is the sole cause for defective RPE and photoreceptor cell death rather than any other unknown mutation occurring in the ENU-induced *r15* mutant line, we generated NHE8 null mutant mice (NHE8-/-) from NHE8 knockout ES cell clones made by the International Knockout Mouse Consortium (www.mousephenotype.org/martsearch_ikmc_project/about/eucomm). The NHE8 gene targeting vector was constructed using the knockout-first-reporter tagged insertion (promotorless cassette) strategy. Insertion of a promotorless cassette into the intron between exons 3 and 4 of the *Slc9a8* gene would result in a truncated NHE8 fusion protein, which expresses only the N-terminal 92 amino acids (encoded by the first three exons) followed by the inserted LacZ+neo in the NHE8 knockout mice.

**Figure 4:**
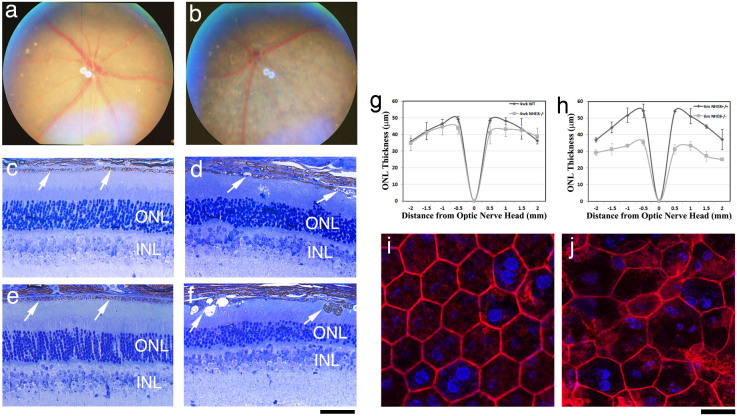
NHE8 knockout mice display retinal phenotypes similar to the *r15* mutation. Fundus photos of 2-month old wild-type (a) and NHE8-/- (b) littermates. The NHE8-/- fundus reveals depigmented patches, similar to that observed in the *r15* mutant mice. Histological sections of 4-week old wild-type (c) and NHE8-/- (d) littermates as well as 6-month old wild-type (e) and NHE8-/- (f) littermates. The NHE8 knockout RPEs displayed vacuoles and abnormal pigmented cellular structures (d,f, indicated by white arrows). At the age of 6 months, the wild-type control had 10–12 outer nuclei layers (ONL) of photoreceptor cells, while NHE8 knockout had only 5–7 layers. Spider graphs show ONL thickness in wild-type (black lines, n = 3) and NHE8-/- mice (grey lines, n = 3) at the ages of 4 weeks (g) and 6 months (h). Phalloidin stained images show typical pentagonal/hexagonal cell shape with intact F-actin network of flat-mount RPE in 1-month-old wild-type mice (i), while variable cell size and abnormal cell shape with disrupted F-actin network of flat-mount RPE in NHE8-/- (j) littermates. Scale bars for panels c–f, 50 μm; for panels i and j, 20 μm.

Similar to the recessive retinal phenotypes in *r15* (NHE8-M120K) homozygous mutant mice, NHE8 knockout mice displayed depigmented patches in the fundus photo (Fig. 4b), disrupted RPE layer with vacuoles (Fig. 4d,f), irregularly sized and shaped RPE cells (Fig. 4j), and an obvious loss of photoreceptor cells at the age of 6 months but not 4 weeks (Fig. 4d,f). Heterozygous NHE8+/- mice had no obvious retinal phenotypes (data not shown). The ONL thickness was measured in wild-type (WT) and NHE8-/- mutant retinas at the ages of 4 weeks and 6 months (Fig. 4g,h). At 4 weeks of age, there is no significant difference in ONL thickness between the control and NHE8-/- mice (p > 0.5); 6-month old NHE8-/- mutant mice display significantly reduced ONL thickness (p < 0.05). Since the *r15* mutant and NHE8 knockout mice display similar recessive retinal phenotypes, NHE8-M120K is likely a dysfunctional point mutation.

**Figure 5:**
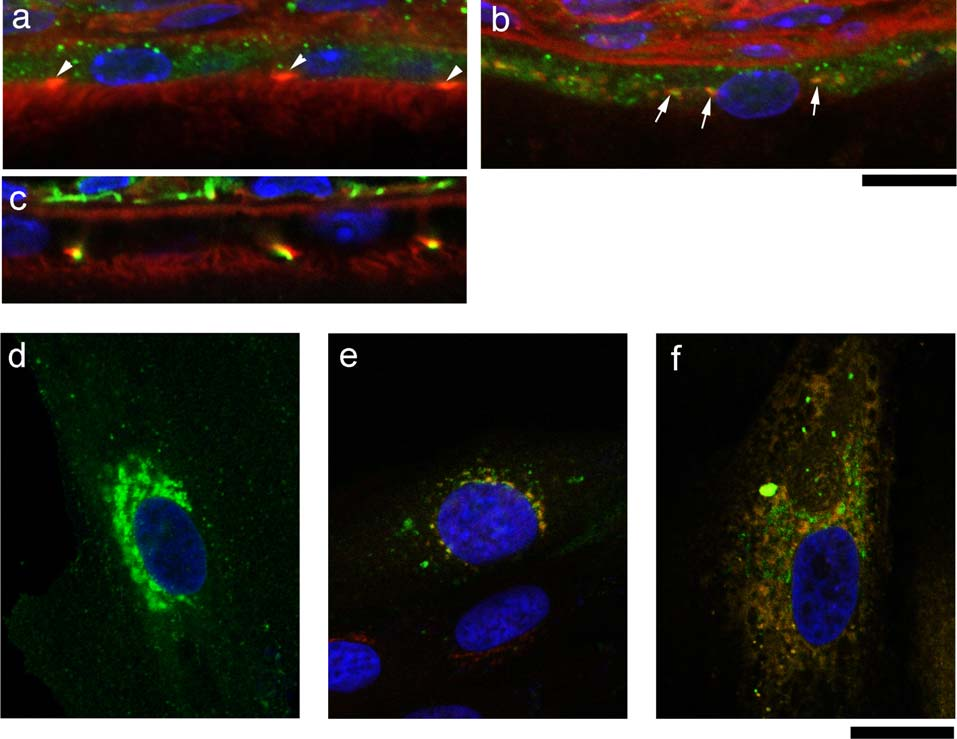
The NHE8 protein is associated with the Golgi complex and the intracellular vesicles. Fluorescent staining results reveal that NHE8 proteins (green) display intracellular vesicular-like signals (a), white arrowheads indicate enriched F-actin (red) at apical/lateral tight junctions. NHE8 proteins (green) are partly colocalized with giantin protein (red, a marker for the Golgi complex) in wild-type RPE, indicated by white arrows (b). Tight junctions labeled by anti-ZO-1 antibody (green) are colocalized with F-actin (c). Scale bars for panels a–c, 10 μm. Human ARPE-19 cells were infected with AAV expressing wild-type NHE8-SE pHluorin, and cells were fixed for staining with antibodies for Golgi and ER; NHE8-SE pHluorin expression (green) displays a more prominent perinuclear pattern as well as vesicular distribution throughout the cell (d); staining with anti-TGN46 (red) reveals colocalization of NHE8 and the Golgi complex (e) in the perinuclear region; staining with anti-calreticulin (red) also suggests a colocalization of NHE8 and ER in the cytosol (f). Scale bars for panels d–f, 20 μm.

In order to elucidate the mechanistic role of NHE8, we characterized the localization of NHE8 proteins in the RPE *in vivo*. Immunohistological data showed that NHE8 proteins displayed an intracellular vesicular expression pattern (Fig. 5a) in RPE cells; NHE8 colocalized with giantin (a marker for the Golgi complex) as well as expressed in other intracellular vesicles, but not at RPE cell boundaries (Fig. 5a,b). Tight junctions labeled by ZO-1 antibody (green) were colocalized with F-actin in RPE cells (Fig. 5c).

Since a previous work reported that NHE8 played a key role in the control of protein trafficking in cultured HeLa M-cells *in vitro*^24^, we investigated whether NHE8 played an important role in the protein trafficking needed for RPE cell polarity. We examined the distribution of NHE8 delivered by Adeno-associated virus (AAV) in human ARPE-19 cells *in vitro*. NHE8-SE pHluorin proteins, expressed from AAV5, were predominantly present in vesicular intracellular structures of ARPE-19 cells (Fig. 5d), and were colocalized with the Golgi marker TGN46 (Fig. 5e) and the endoplasmic reticulum (ER) marker calreticulin (Fig. 5f).

**Figure 6:**
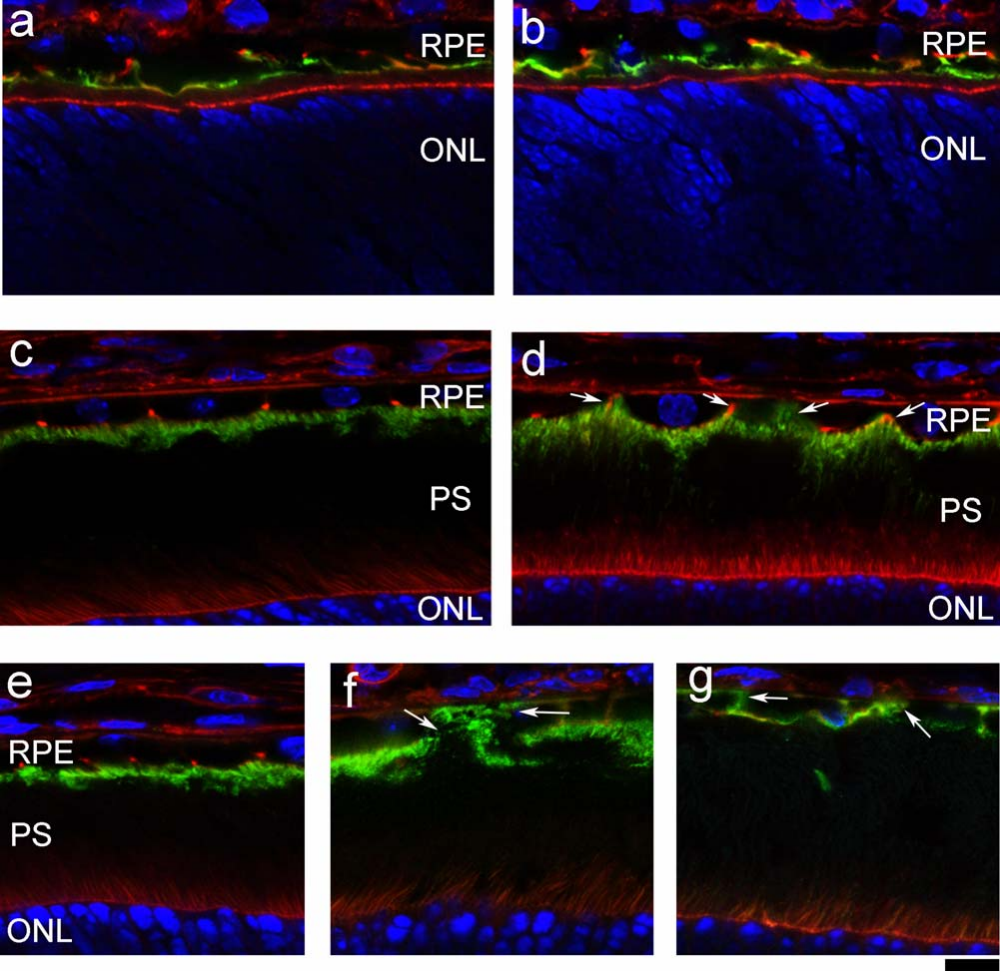
NHE8 is important for the polarity of ezrin expression in RPE cells. Immunostaining of ezrin proteins (green, a marker for the apical side of RPE) and F-actin (red) in wild-type (a) and NHE8-/- retinal sections (b) at the age of postnatal day 7 (P7) show very similar ezrin distribution in the apical RPE as well as some lateral sides. At P14, ezrin distribution is mostly restricted to the apical side of RPE in the wild-type (c), while some extended ezrin expression in the lateral sides towards the basal sides is observed in the NHE8-/- retina (d, white arrows). Panels e–g show the immunodistribution of ezrin proteins in the wild-type (e), *r15* mutant (f) and NHE8-/- (g) retinas of mice at the age of 4 weeks. White arrows indicate mislocalized ezrin at basal and lateral sides of *r15* and NHE8-/- RPEs. Scale bar, 10 μm.

We further examined the expression of ezrin, a suitable apical marker for monitoring RPE cell polarity, even though it could be detected at a very low level in the basal-lateral sides. As expected, ezrin proteins were predominantly located at the apical sides of the RPE in the wild-type mouse retina (Fig. 6a,c,e). However, aberrant lateral-basal distribution of ezrin proteins in RPE was detected as early as two weeks of age in NIH8-/- mice (Fig. 6d). Ezrin also appeared in apical, lateral and basal sides of RPE in 4-week old NHE8-M120K (Fig. 6f) and NHE8-/- (Fig. 6g) mice. Consistent with previous morphological data (Fig 1,2,4), RPE cell polarity is disrupted in NHE8-M120K and NHE8-/- mutant retinas.

## Discussion

This work demonstrates that NHE8 is essential for the survival of photoreceptor cells, and it plays an important role for the polarity and function of RPE cells. Both NHE8 knockout and NHE8-M120K mutant mice display late-onset loss of photoreceptor cells and impaired RPE cells including altered cell morphology, mislocalized ezrin proteins and a loss of apical microvilli. Since RPE cell defects occur before the loss of photoreceptor cells in both NHE8 mutant mouse lines, the slow loss of photoreceptors is likely a consequence of impaired RPE function. Based on the fact that NHE8 proteins are localized in protein trafficking compartments such as the Golgi, ER and other unknown intracellular vesicles in RPE, we hypothesize that NHE8 may regulate pH homeostasis in the protein trafficking pathway and/or in intracellular vesicles. However, further studies will be needed to test this hypothesis. The underlying mechanism for altered distribution of the cell polarity marker ezrin is unknown; it is possible that NHE8 loss-of-function mutations perturb ezrin's trafficking, sorting and/or targeting to its final destination. A loss of NHE8 function alone is sufficient to impair RPE and leads to the slow loss of photoreceptor cells in mice. The presence of other NHE isoforms and other pH regulators are unable to compensate the functional loss of NHE8 in the retina. It will be important to investigate whether or not NHE8 plays an essential function in humans. These NHE8 mutant mice provide an excellent model system to study intracellular pH homeostasis that is largely unknown in the RPE.

All key functions of the RPE rely on its cell polarity^6^, such as precisely localized specific receptors for phagocytosis and polarized structures to regulate the transport of nutrients and waste products^3^,^10^,^25^,^26^,^27^. Ezrin is an apical marker of the RPE due to its high abundance in apical microvilli, even though very low levels of ezrin are present in basal infoldings of RPE^28^,^29^,^30^. Ezrin is needed for the proper formation of microvilli and basal infoldings of RPE^31^ and for the function of NHE3^32^. Thus, NHE8 mutations will cause many indirect consequences to impair the function of RPE. Transporters such as proton-coupled monocarboxylate transporters (MCTs) are specifically positioned apically or basolaterally to facilitate the transport of lactate and H^+^ out of the retina to regulate pH, ion homeostasis and the high-energy demands of photoreceptor cells^2^,^33^,^34^. MCT3 and MCT4 harbor dominant sorting information for the basolateral membrane^35^. It will be interesting to study whether or how MCTs are affected in NHE8 mutations.

The polarized trafficking machinery, composed of secretory organelles (ER and Golgi complex) and endosomal compartments, is critical for the generation and maintenance of the asymmetric distribution of plasma membrane proteins^36^. The trans-Golgi network (TGN) is where cargoes undergo sorting, packaging and delivery to different destinations such as the plasma membrane, the endosomes, or secretory granules. Each of these transport routes probably uses a specialized and dedicated machinery^37^. However, the underlying mechanism for how polarized protein trafficking contributes to RPE plasma membrane polarity is still poorly understood^6^. NHE8 is known to contribute to the maintenance of the unique acidic pH values of the Golgi and post-Golgi compartments in cultured cells^38^. The pH values gradually decrease from ~7 to ~5, starting in ER, Golgi, TGN and secretory granules, and from early and late endosomes (pH ~ 6.5) to lysosomes (pH ~ 4.5) in general^39^. This progressive acidification is essential for post-translational modifications, including sorting of newly synthesized proteins into the secretory pathway. Studies from cultured Hela M-cells *in vitro* have reported that NHE8 plays a key role in the control of protein trafficking and endosome morphology^24^. The majority of epitope-tagged NHE8 was found in the trans-Golgi network of HeLa M-cells, but a proportion was also localized to multivesicular bodies (MVBs). Depletion of endogenous NHE8 or overexpression of a nonfunctional point mutant protein NHE8-E225Q affected endosome morphology but did not affect the overall pH inside dense MVBs. Our unpublished data show that endosome marker EEA1 seems not colocalize with NHE8 in RPE cells. So the function of NHE8 in RPE cells likely differs from its role in HeLa M-cells. NHE8 is also known to be an apically expressed membrane protein in intestinal epithelial cells for controlling sodium absorption and bicarbonate secretion in the intestine^22^. However, NHE8 protein is hardly detected in either the apical or basolateral membrane of RPE (Fig. 5a,b). Intracellular pH changes in polarized protein trafficking of RPE have not been investigated *in vivo* nor in cultured RPE cells *in vitro*. NHE8 mutants may be useful for exploring pH homeostasis in the polarized trafficking machinery needed for RPE cell polarity as well as the luminal pH regulation in the secretory pathway of RPE during development and aging.

## Methods

### Animals

All studies and examinations were conducted in accordance with a protocol approved by the Animal Care and Use Committee (ACUC) at University of California, Berkeley. ENU-mutagenesis and breeding of the *r15* mice were performed as previously described^40^,^41^. NHE8 knockout mice were acquired from the Mutant Mouse Regional Resource Center (MMRRC) at UC Davis. The targeting vector was designed to insert a promotorless cassette into the intron between exons 3 and 4 of the *Slc9a8* gene, which would result in a truncated NHE8 fusion protein that includes the N-terminal peptide (encoded by the first three exons) and the inserted LacZ and neo. For genotyping, a primer pair of forward 5′-GAA GTA GGT CTC TCA CTG G and reverse 5′-AAT CTT GAC CAT AGC TGT CCT CCA CC generates a wild-type PCR fragment (~500 bp) and a larger knockout band (~575 bp) due to the insertion of a loxp site in the targeting vector.

### Fundus examination and histology

Fundus examination and retinal histology were performed as previously described^41^.

### Genomic linkage analysis

A female founder was identified from a screen of ENU-induced F3 mutagenized mice by fundus examination, and chromosome mapping was performed according to previously described methods^20^,^21^. Homozygous *r15* mutant mice in the C57BL/6J strain background were mated with wild-type C3A.BLiA-*Pde6b*^+^/J mice to produce G1 hybrid mice; G1 hybrids were further mated with homozygous r15 mutant mice to produce second-generation (G2) mice. The G2 mice were examined for retinal phenotype, and genomic DNA samples were extracted from tail snips for genome-wide linkage analysis using a total of 59 microsatellite markers. After the chromosomal linkage was identified, we further performed fine mapping. Marker sequence information (D2Mit51, D2Mit145, D2Mit229, D2Mit344 and D2Mit200) was obtained from the Ensembl Mouse database.

### DNA sequencing

Retinas were dissected from homozygous *r15* mutant mice and total RNA was isolated using the TRIzol® Reagent (Invitrogen Life Technologies). The Superscript™ First-Strand Synthesis System for RT-PCR kit (Invitrogen Life Technologies) was used to synthesize cDNAs. The coding region of the NHE8 gene was amplified by various primer pairs with Platinum® pfx DNA polymerase (Invitrogen Life Technologies). PCR fragments with overlapping regions were sequenced at the UC Berkeley DNA sequencing facility.

### Immunofluorescence studies

Immunofluorescent staining of retinal frozen sections was performed as previously described^41^. For staining RPE whole mount, mouse eyes were fixed in 4% formaldehyde/PBS, the cornea and lens were removed, followed by antibody staining and the remaining eye cups were flat-mounted for imaging. A rabbit polyclonal antibody for NHE8 was generated using the C-terminal 14-amino acid peptide as immunogen; other antibodies used for immunostaining were: RPE65 mouse monoclonal antibody (generously provided by Dr. Debra Thompson, University of Michigan), ezrin rabbit polyclonal antibody (Cell Signaling Technology), ZO-1 rabbit polyclonal antibody (Invitrogen Life Technologies), giantin mouse monoclonal antibody^42^, TGN46 rabbit polyclonal antibody (Millipore), calreticulin rabbit polyclonal antibody (Stressgen Biotechnologies) and rhodamine phalloidin (Molecular Probes). All images were collected by a Zeiss LSM700 confocal microscope.

### Measurement of the ONL thickness

Retinal histology sections cut through optic nerve head and the ora serrate were imaged, and the ONL thickness was measured using the ImageJ program. Retinal sections from 3 mice for each genotype at each age were measured, and the average values and standard deviations (SD) were graphed. Student's T-tests were used to determine the statistical significance between controls and mutants.

### AAV5-NHE8-SE pHluorin infection

To make AAV5-smCBA-NHE8-SE pHluorin, we first constructed the expression plasmid. SE pHluorin was cloned into pEGFP N1 vector (Clontech laboratories) to replaced EGFP by the BamHI-SE pHluorin-NotI fragment to obtain pHluorin pN1 vector; the EcoRI-NHE8-BamHI fragmet was then subcloned into pHluorin pN1 vector to generate pN1-CMV-NHE8-SE pHluorin vector; NHE8-SE pHluorin fragment was cut with XhoI and NotI from the pN1-CMV-NHE8-SE pHluorin vector to replace VAMP2-SE pHluorin in the pTR-smCBA-VAMP2-SE pHluorin plasmid described below to obtain final pTR-smCBA-NHE8-SE pHluorin plasmid. The pTR-smCBA-VAMP2-SE pHluorin plasmid was constructed by replacing the hGFP fragment of the pTR-smCBA-hGFP vector with a VAMP2-pHluorin PCR fragment with XhoI and NotI restriction sites. AAV5-smCBA-NHE8-SE pHluorin was generated with the pTR-smCBA-NHE8-SE pHluorin plasmid^43^,^44^.

For AAV infection, human ARPE-19 cells were plated on 35 mm glass bottom plates, and the cells were 50% confluent at the time of infection. The cells were infected with 1 × 10^12^ vector genomes (vg)/ml AAV5-smCBA-NHE8-SE-pHluorin; 72 hours later, infected cells were fixed with 4% formaldehyde/PBS for 10 minutes, followed by antibody staining and confocal imaging.

## Author Contributions

C.X. and X.G. are responsible for conceptual idea. C.X., H.L., D.C., F.T., B.C. and M.L. conducted the experiments. C.X. and X.G. wrote the manuscript.
